# Supplementary material for: Employee engagement, perceived organizational support, and job performance of medical staff at the Cape Coast Teaching Hospital
Source: PLoS One. 2024 Dec 30;19(12):e0315451. doi: 10.1371/journal.pone.0315451 (PMC11684727; doi:10.1371/journal.pone.0315451)
Supplement: Attachment — Submitted filename: Response to Reviewers 1.docx [file pone.0315451.s002.docx]

**Response to Reviewers**

Thank you for your valuable feedback. We have modified the manuscript according to your opinions and answered the questions raised by reviewers point by point all with the red context like here. It is appreciated to be recognized by reviewers.

| Reviewer Content | Response |
| --- | --- |
| Reviewer 1 |  |
| 1. This research is very simple, you have shown the position of this research compared to previous research. My question is, what is the urgency of this research...? | The urgency of the study has been added as the last but one paragraph in the introduction on page 3 |
| 2. Some of the references used still use old years. | Current references have been included in the manuscript in the second line of the first paragraph on page 1. For instance; Yildiz, B., & Yildiz, T. (2022). A systematic review and meta-analytical synthesis of the relationship between work engagement and job satisfaction in nurses. Perspectives in Psychiatric Care, 58(4).  Wei, H., Sewell, K. A., Woody, G., & Rose, M. A. (2018). The state of the science of nurse work environments in the United States: A systematic review. International journal of nursing sciences, 5(3), 287-300. |
| 3. The author must explain why you chose the Cape Coast Teaching Hospital (CCTH) as the research object? in the methodology section | Justification for choosing CCTH has been added as the last paragraph on page 9 (the study organisation, target population and sampling procedures). |
| 4. You need to explain what the shortcomings of this research are to provide material for further research development | Limitations of the study have been added to the last paragraph of the (conclusion, limitation and suggestion for future study) on page 24. |
|  |  |
| Reviewer 2 |  |
| 1. In the introduction section, I think it would be a good idea to elaborate on the practical implications of the research. Including a brief discussion on how the findings might benefit hospital management and policy-making in the healthcare sector. Also, adding recent statistics on employee engagement and job performance in healthcare could provide better insights on the relevance of this research. | A recent statistic on engagement has been added to the first paragraph of the introduction on page 1.  Again, the benefits of the study’s findings has been added to the last paragraph of the introduction on page 3. |
| 2. In the literature review, it is very important to cover relevant theories and previous studies. To improve this section, consider adding a comparative analysis of similar studies conducted in different cultural or regional contexts. This would highlight the uniqueness and relevance of the current study. I suggest adding a conceptual mal or diagram to summarize key findings of the existing literature as well as an author's table where you explain the most relevant research on this topic. | The study is underpinned by the social exchange theory which to our understanding has been adequately reviewed. Notwithstanding, we have added some few materials to the review. See first paragraph on page 5.  Again, we have empirically reviewed a number of papers from diverse situations and circumstance to buttress the issues of cultural/diversification in relation to the topic. |
| 3. The methodology is robust and well-detailed, but it could benefit from a more in-depth discussion on the selection criteria for participants and potential biases. Adding a section on how the data collection process was standardized across different respondents would improve transparency. I also suggest to include an explanatory diagram explaining each step of your research. | A discussion on the selection criteria for participants and potential biases has been added as the last paragraph of (the study organization, population and sampling) on page 9. That is a proportion to size of 25% was used to select from each stratum.  A section on data collection has been added to the methods on page 11. |
| 4. The results section is clearly presented and supported by appropriate statistical analysis. However, to enhance the depth of the analysis, consider incorporating Importance-Performance Map Analysis (IPMA) and Multi-Group Analysis (MGA) as part of the sensitivity analysis. These additions would provide the best understanding of the data and highlight key areas for improvement or further investigation. Adding visual aids like graphs and charts also can explain findings and recommendations in a better way. | Two diagrams have been added to the analysis section (IPMA and Slope Analysis) (Fig. 3 and Fig.4) |
| 5. The conclusions are well-supported by the data, but the section could be strengthened by including more specific recommendations for hospital management and contrasting findings with previous studies. Mention also, how each of the objectives of this research was completed, it could include a table or a diagram to explain this. | Recommendation for hospital management has been added to the writeup (Conclusion, Limitations and Suggestions for Future Research) on page 25. |
